# Immature and mature pathological tau forms in the retina of MCI and AD patients correlate with brain tauopathy

**DOI:** 10.1002/alz.087770

**Published:** 2025-01-03

**Authors:** Dieu‐Trang Fuchs, Haoshen Shi, Nazanin Mirzaei, Yosef Koronyo, Miyah R Davis, Edward K Robinson, Altan Rentsendorj, Lon S. S. Schneider, Debra Hawes, Julie A. Schneider, Keith L Black, Rakez Kayed, Maj‐Linda B Selenica, Daniel C Lee, Maya Koronyo‐Hamaoui

**Affiliations:** ^1^ Department of Neurosurgery, Maxine Dunitz Neurosurgical Research Institute, Cedars‐Sinai Medical Center, Los Angeles, CA USA; ^2^ Neurosurgery Department, Cedars‐Sinai Medical Center, Los Angeles, CA USA; ^3^ Department of Psychiatry and Behavioral Sciences, Keck School of Medicine, University of Southern California, Los Angeles, CA USA; ^4^ Department of Pathology, Keck School of Medicine, University of Southern California, Los Angeles, CA USA; ^5^ Rush Alzheimer’s Disease Center, Rush University Medical Center, Chicago, IL USA; ^6^ University of Texas Medical Branch, Galveston, TX USA; ^7^ Sanders Brown Center on Aging, College of Medicine, University of Kentucky, Lexington, KY USA

## Abstract

**Background:**

This study identifies and quantifies diverse pathological tau forms in the retina at both early and advanced stages of Alzheimer’s disease (AD) and assesses their correlation with disease status. In the pathogenesis of AD, the tau protein undergoes post‐translational modifications, including hyperphosphorylation (p‐tau). As the disease progresses, pathological tau can propagate as oligomers, aggregate into fibrils, and paired helical filaments (PHF), and ultimately form intraneuronal neurofibrillary tangles (NFTs). Previously, increased p‐tau and other abnormal forms were detected in postmortem retinas of AD patients; however, their presence and levels at earlier stages – in mild cognitively impaired (MCI due to AD) patients – and their association with brain pathology remain undefined.

**Method:**

In this study, we acquired 75 postmortem human eyes and 39 paired brains. Retinal cross‐sections in predefined geometric regions were prepared from 34 AD and 11 MCI patients, and 30 age‐ and sex‐matched cognitively normal controls. Histological analyses involved Bielschowsky silver staining and immunostaining of brains and retinas with antibodies for tau (pS396, AT8, AT100, CitR_209_, T22, PHF‐1, and MC‐1). Stereological examination and quantification were conducted, and correlations were determined with brain pathology and cognition. P‐tau forms were also determined by NanoString GeoMx digital spatial profiling.

**Result:**

Our immunohistochemical examination revealed significant upregulation of immature hyper‐citrullinated (CitR_209_) and p‐tau forms, propagating tau oligomers, as well as mature PHF‐tau and NFT forms in the retinas of patients with early‐AD (MCI due to AD) and AD‐dementia stages. Interestingly, retinal PHF‐tau accumulated only in the AD‐dementia stage. GeoMx spatial profiling analyses revealed site‐specific increases in various p‐tau epitopes in AD, particularly at the early MCI stage. Tau oligomers had the largest accumulations in MCI and, moreover, AD retinas, showing a strong correlation to Braak staging, representing the spread of tauopathy across brain regions during disease progression. Strong correlations were also found between retinal tauopathy and brain NFTs, ABC, and MMSE severity scores.

**Conclusion:**

Our data demonstrate that most forms of retinal tauopathy are increased in early AD and correlate with one or more AD neuropathology and cognitive parameters, encouraging the development of retinal tauopathy imaging for AD detection and monitoring disease progression.

